# Degradation of Triclosan and Carbamazepine in Two Agricultural and Garden Soils with Different Textures Amended with Composted Sewage Sludge

**DOI:** 10.3390/ijerph15112557

**Published:** 2018-11-14

**Authors:** Yanqiu Shao, Kai Yang, Rongchang Jia, Chao Tian, Ying Zhu

**Affiliations:** 1 Advanced Materials Institute, Qilu University of Technology (Shandong Academy of Sciences), Jinan 250014, China; shaoyq@sdas.org (Y.S.); yangk@sdas.org (K.Y.); tianchao@sdas.org (C.T.); 2 Laboratory for Earth Surface Processes, College of Urban and Environmental Sciences, Peking University, Beijing 100871, China; 3 Chemical Technology Academy of Shandong, Qingdao University of Science and Technology, Jinan 250014, China; 18668968788@163.com

**Keywords:** composted sewage sludge, PPCPs degradation, triclosan, carbamazepine, soil properties, enzyme activities

## Abstract

Composted sewage sludge (CSS) has been extensively used in agriculture and landscaping, offering a practical solution for waste disposal. However, some pharmaceutical and personal care products (PPCPs) like triclosan (TCS) and carbamazepine (CBZ) have restricted its land application. In this study, CSS was added to agricultural soil and garden soil at 0%, 5%, 10%, and 25% (*w*/*w* soil), and 4 mL of TCS and CBZ stock solution (1000 mg/L in methanol) was spiked into soil amended with CSS of each bottle to arrive at the concentration of 10 mg/kg. Samples were then collected after incubation for 120 days and analyzed for concentrations and half-life (*t*_1/2_) of TCS and CBZ, and soil physicochemical properties, together with enzyme activities. The results showed that TCS was degraded completely during the incubation period. In contrast, only about 5.82–21.43% CBZ was degraded. CSS amendment inhibited TCS and CBZ degradation and prolonged *t*_1/2_ compared to the control, and the *t*_1/2_ of TCS and CBZ increased with CSS addition amount in all treatments except for CBZ in the garden soil amended with 10% CSS. Correlation studies showed a significantly positive relationship between *t*_1/2_ of TCS and CBZ and total organic carbon (TOC), while a significantly negative relationship between *t*_1/2_ of the two PPCPs and pH was observed. Alkaline phosphatase showed a significantly negative relationship with the C_t_/C_0_ of TCS in garden soil amended with 25% CSS and CBZ in the control. The urease activity was negatively correlated with the C_t_/C_0_ of TCS in 10% and 25% CSS treatments and CBZ in 10% CSS treatment for garden soil.

## 1. Introduction

Pharmaceuticals and personal care products (PPCPs) as emerging organic pollutants have received more and more attention during the past decades, due to their frequent detection in the soil environment and their long-term potential risks to soil organisms and aquatic systems [1,2]. PPCPs as essential components of modern life are frequently consumed by humans and animals. After being excreted or washed out of the body, PPCPs can accumulate in the sewage system and are treated in wastewater treatment plants [3]. Incomplete removal and/or degradation of PPCPs in wastewater treatment plants has led to its ubiquity in treated wastewater (ranging from ng to mg L^−1^) and in biosolids (μg to mg kg^−1^) [4,5]. Hence, irrigation with treated wastewater and/or amendment with biosolids can release PPCPs to the environment [6]. Once in the soil, PPCPs may undergo adsorption/desorption [7], transport [8], degradation/transformation [9], accumulation into edible parts or leaching to contaminate groundwater or surface water, constituting a risk for secondary pollution [10]. Also, it can enter human bodies via soil ingestion, soil dust inhalation, and soil dermal contact, therefore causing severe health effects [11,12].

Among many PPCPs, triclosan (TCS), an anti-bacterial agent, has been found in numerous household and healthcare products like soaps, toothpaste, cosmetics, shampoo, and textiles. TCS has been designated as one of the high-production-volume chemicals by the U.S. Environmental Protection Agency (U.S. EPA) [10,13,14] and is frequently detected in biosolids in the order of 20 to 40 mg kg^−1^ [4,15]. Researches have shown that TCS may induce developmental, carcinogenic, or other chronic toxicities to human and other non-target organisms [16,17,18]. Carbamazepine (CBZ), an antiepileptic drug, is known as a relatively persistent compound in the environment. It is one of the most frequently detected PPCPs in the wastewater effluent and biosolids [3,19]. CBZ has been reported to occur in treated wastewater effluents up to 6.3 μg L^−1^ and in biosolids up to 258 μg kg^−1^ [4,5]. In addition, CBZ-related teratogenic effects have been observed clinically [5].

As one of the main carriers of PPCPs into soil, biosolids have been widely used in foreign countries. For example, the production of biosolids in the USA and Europe is about 7.2 and 4.7 million tons of dry materials per year, respectively [20]. Approximately 50% of biosolids are applied as an organic amendment to agricultural lands in Europe. In the USA, biosolids are typically applied annually or up to three times per year [6]. While in China, composted sludge application has not been widely used. In fact, it has been strictly controlled for agricultural processes, because elevated contents of metals and organic pollutants in biosolids may cause negative impacts on water, soil, and air. However, in landscaping, it is prevalent for its rich nutrients. The application of biosolids to soils can greatly alter soil physicochemical properties. It can increase soil organic matter (OM) content, water-holding capacity, porosity, nutrient level, and microbial number, and thereby promote plant growth and yield [10,21,22].

However, biosolids amendment tends to result in the accumulation of PPCPs in soil to some extent. PPCPs in soil may be degraded or transformed as a result of abiotic reactions. Previous studies have confirmed that the degradation PPCPs was closely correlated with soil properties [7,23]. When biosolids are used to amend soil, the degradation of PPCPs in soil is inevitably affected by the biosolids through the effects the biosolids have on the soil properties. It was reported that biosolids amendment generally inhibited the degradation of PPCPs [5,24], therefore prolonging their persistence in soil. This was mainly due to the increase in soil organic matter content by biosolids which may increase the sorption of PPCPs to soil due to decreased bioavailability [5,10]. Fu et al. [10] and Williams et al. [25] found that the sorption of TCS and CBZ were enhanced in biosolids amendment soil. In contrast, it was also confirmed that biosolids amendment had no significant effects on the degradation of TCS and triclocarban [26]. The inconsistent degradation behaviors of PPCPs may differ with test soils and composted sewage sludges (CSS). Different soils amended with different CSS will lead to various total organic carbon, pH, clay content, cation exchange capacity, and biological properties in soil [10,26].

To date, a number of studies have considered the degradation or persistence of TCS and CBZ in soil with or without biosolids amendment. Most of them focused on the degradation of PPCPs in biosolids-amended agricultural soils [10,27] while a few studies used urban garden soils. The correlation between soil organic matter and *t*_1/2_ of PPCPs in agricultural soil was frequently analyzed [1,10]. However, regarding pH, a correlation study between it and *t*_1/2_ of PPCPs was less involved. Additionally, more studies focused on the effects of PPCPs on enzyme activities [28,29], but less involved the relationship between them and the degradation *t*_1/2_ of PPCPs.

The aims of the present study were to: (1) evaluate the degradation of TCS and CBZ in agricultural and garden soils amended with CSS, determining the impact of CSS on the degradation of TCS and CBZ; (2) analyze the relationships between *t*_1/2_ of TCS and CBZ and soil TOC, pH, and soil enzyme activities.

## 2. Methods and Materials

### 2.1. Soils and CSS

Two types of soil were selected as the test soil. The agricultural soil was collected from a farmland with a wheat–corn rotation system, located in Jiyang County of Jinan, Shandong Province of China (about 0.33 ha, N 36°46′ 55 and E 117°0′ 50). Jiyang County has a population of 552,400 and is located in the alluvial plain of the Yellow River, in which agriculture is the main industry and there is no pharmaceutical factory. About 10-kg composite samples were obtained from the 0–0.2 m surface layer by mixing ten random pure samples from each regional sampling point. The garden soil was collected from the 0–0.1 m surface layer of an existing typical recreational garden (approximately 0.35 ha, N 36°38′38.66″, E 117°2′29.69″) in the Shandong Academy of Sciences in Jinan, using a plastic trowel. The CSS was supplied by Guangdong Huayang Environmental Science and Technology Co., Ltd., in Zhaoqing, China. The CSS has been used in commercial tree planting to supply nutrients and organic matter for years. The soil and CSS were air-dried, gently crushed, and passed through a 2-mm stainless steel sieve to remove gravels, plant materials, and other debris.

### 2.2. Incubation Experiments

The CSS was added to the agricultural soil and the garden soil at 0%, 5%, 10%, 25% on a dry mass basis (*w*/*w*), which was labelled as A0, A5, A10, A25, G0, G5, G10, and G25, respectively. An aliquot of 400 g (dry weight) soil and CSS mixture was placed in a 600 mL plastic bottle. First, the soil moisture was adjusted to 30% of the soil water holding capacity using deionized water. After a 7 day pre-incubation, 40 g of soil sample of each bottle was taken and spiked with 4 mL of CBZ and TCS mixed solution (1000 mg/L in methanol). Then, the plastic bottles were left open in a fume hood until the solvent was evaporated. Afterward, the 40 g of contaminated soil was mixed thoroughly with the remaining soil using a stainless steel spatula, and distilled water was added to adjust the soil moisture to 60% of the water holding capacity. The soil bottles (120 mm in height, 80 mm in diameter) with air holes on their top cap were placed in a 25 °C incubator and kept in the dark. The samples were collected at the 1st, 10th, 30th, 60th, and 120th day, and one part of the collected soil was freeze-dried for PPCPs determination. Another part was kept at 4 °C for enzyme activity determination, and the remaining soil was air-dried for physical and chemical properties determination.

### 2.3. Laboratory Analysis

#### 2.3.1. Physicochemical Analysis

Sample pH and electrical conductivity (EC) were determined by the 1:5 solid/water (*w*/*v*) suspension method using a calibrated pH meter (PHS-3E, Rex, Shanghai, China) and an EC meter (DDS-307A, Rex, Shanghai, China) [30]. Cation exchange capacity (CEC) was determined by the ammonium acetate titration method [30]. TOC content was determined by the potassium dichromate titration method [30]. Particle size distribution was determined by the laser diffraction method using a particle size analyzer (Mastersizer 2000, Malvern, Malvern, UK) [31,32].

#### 2.3.2. PPCPs Determination

Freeze-dried soil (2 g) was added to a centrifuge tube and supplemented with 5 mL of methanol. The tube was securely closed, agitated for 2 min on a vortex mixer, and extracted for 20 min with ultrasonication, then centrifuged for 10 min at 4000 rpm. Soil was sequentially extracted a further two times, the supernatants pooled and passed through a 0.22-μm PTFE filter membrane. All final samples were stored at −20 °C prior to instrumental analysis. Instrumental analysis was performed on a Ultimate 3000 high-performance liquid chromatography system (Dionex, Sunnyvale CA, USA). The chromatographic column was a C18 reversed phase column (4.6 × 150 mm, 5 μm) with 80% methanol in water and water as the mobile phases B and A, respectively, and the system was programmed (with respect to mobile phase B) for 0–15 min, at a flow rate of 0.8 mL/min. The injection volume was 10 μL and the column temperature was 30 °C. Quality control for PPCPs extraction tests was assessed by the use of a soil spiked with 4 mL of PPCPs (1000 mg/L in methanol), and duplicate samples. The recovery rates for TCS and CBZ in two soils varied from 87% to 97%.

#### 2.3.3. Soil Enzyme Determination

Dehydrogenase was determined by measuring the rate of reduction of 2,3,5-triphenyltetrazolium chloride (TTC) as a substrate, which was described by Serra-Wittling et al. [33]. Moist soil (4 g) in 50 mL plastic bottles were treated with 2 mL of 1% TTC and 2 mL of 1% glucose solution, and then incubated at 37 °C for 24 h in darkness. At the end of incubation, the 1,3,5-Triphenyltetrazolium formazan (TPF) with reddish color formed by the reduction of TTC was extracted with 10 mL methanol for three to four times to ensure the complete removal of TPF. The filtrate was then diluted with additional methanol to a final volume of 50 mL, and measured absorbance at 485 nm.

Soil alkaline phosphatase enzyme activity was determined using the method described by Lu [34]. Briefly, 1 g fresh soil (<2 mm) in a 50 mL triangular flask was mixed with 0.2 mL toluene, 4 mL (pH = 11) buffer solution and 1 mL 4-nitrophenyl phosphate disodium (0.05 mol/L) and incubated at 37 °C for 1 h. After that, 1 mL of CaCl_2_ (0.5 mol/L) and 4 mL of NaOH (0.5 mol/L) were added, then the mixture was filtered. The five times dilution of the filtrate was conducted before it was quantified with the spectrophotometer at 420 nm. Blanks without the incubation were carried out in the same manner.

Soil urease activity was determined by the following method described by Guan et al. [35]. Air-dried soil samples (5.0 g) in 50 mL triangular flasks were mixed with 1 mL toluene for 15 min before 10 mL of 10% urea and 20 mL of citrate buffer (pH = 6.7) were added. The samples were placed in an incubator at 37 C for 24 h. After filtration, 3 mL filtrate was immediately transferred into a 50 mL flask and distilled water was added to 20 mL, then 4 mL sodium phenate (1.35 M) and 3 mL sodium hypochlorite (active chlorine 0.9%) was added successively, at the same time, shaking well with each addition. After 20 min coloration, each sample was diluted by distilled water to 50 mL and the concentration of NH_4_^+^ ions produced from urea hydrolysis was determined at 578 nm as a blue-colored complex to represent urease activity.

Catalase activity was assayed according to Guan et al. [35]. It was determined by mixing 2 g air-dried soil with 40 mL distilled water and 5 mL of 0.3% H_2_O_2_ and shaking for 20 mins. After that, 5 mL 1.5 mol/L H_2_SO_4_ was added to stop the enzymatic activity. The solution was filtered and a 25 mL aliquot was titrated with 0.01 M KMnO_4_. A blank was conducted with a mix of 40 mL of distilled water, 5 mL of 0.3% H_2_O_2_, and 5 mL of 1.5 M H_2_SO_4_, and 25 mL of this mixture was titrated with KMnO_4_.

### 2.4. Data Analysis

The results obtained in the biodegradation assays were fitted to the first order kinetic model described by Equation (1):

(1) $$C_{t}=C_{0}\times e^{-kt}\;$$

where *C*_0_ and *C_t_* are the concentrations of the target pollutants at time 0 and *t* (ug/kg); *k* is the first order degradation rate constant (1/day for the biodegradation tests), and *t* is the experimental time (in days for biodegradation experiments) [36]. Since experiments were carried out in triplicate, average degradation rates and standard deviations were obtained.

In the case of biodegradation tests, the half-life was determined for each pollutant in the tested conditions by using Equation (2):

(2) $$\;t_{1/2}=\frac{ln2}{k}\;$$

where *t*_1/2_ is the half-life for each compound under the experimental conditions (days) and *k* is the first-order degradation rate constant (1/day) [36].

Statistical analysis was performed using IBM SPSS Statistics 18 (Armonk, NY, USA) and Excel 2010 (Microsoft, Redmond WA, USA). Correlations between various variables were determined using Pearson’s product moment correlation coefficient (*r*) by Statistical Product and Service Solutions (SPSS) software for the relationships between degradation half-lives of TCS and CBZ and soil properties and enzymatic activities.

## 3. Results and Discussion

### 3.1. Physicochemical Properties

Table 1 shows the physicochemical properties of the agricultural soil, garden soil, and CSS. Agricultural soil was moderately alkaline (pH 8.16), and contained 10.63 g/kg of organic carbon, in which clay content accounted for about 4.7%. Compared to agricultural soil, garden soil was more alkaline, contained less organic carbon and a higher clay content. The CSS was rich in various nutrients, which can alter the physical and chemical properties of the soil. The addition of CSS decreased the pH value of the soil. The pH value gradually decreased upon increasing the CSS. The pH value decreased from 8.16 to 7.73 in the agricultural soil and from 8.41 to 7.75 in the garden soil. The addition amount of CSS was negatively related to pH in the garden soil (r = −0.812, *p* < 0.05), but positively correlated with TOC in the two soils (r = 0.950, *p* < 0.01). In the garden soil, CEC was highest in the 5% CSS treatment, followed by the 25% CSS treatment, and the value in the control was the lowest. The clay content in the agricultural soil increased firstly and then decreased with the increase of CSS added amount. The clay content was highest in 10% CSS treatment.

### 3.2. Degradation of TCS and CBZ

**Figure 1:**
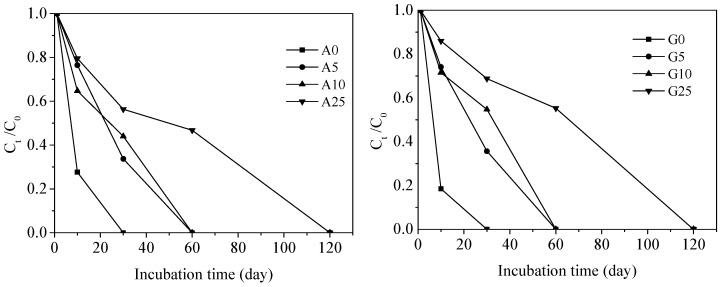
The degradation kinetics of triclosan (TCS) in agricultural and garden soil (A represents agricultural soil, G represents garden soil).

Figure 1 shows the degradation kinetics of TCS in the agricultural soil and garden soil. Regarding the agricultural soil, the total removal of TCS was achieved upon 30 days in the control, whereas in the 5% and 10% CSS treatments, the total degradation of TCS was observed at 60 days. When the CSS application rate was 25%, the degradation rate was lowest, as evidenced by the 120-day total degradation of TCS. With respect to the garden soil, the degradation rate was slightly slower than that for agricultural soil according to the degradation rate constant (*k*) in Table 2, but the complete removal of TCS occurred by the 30th day in the control soil, by the 60th day in the 5% and 10% treatments, and by the 120th day in the 25% treatment. 

As shown in Table 2, the *t*_1/2_ of TCS increased with CSS application rate. The *t*_1/2_ of TCS in the agricultural control and garden control was 4.85 and 3.70 days, respectively. The value increased to 55.45 days in the agricultural soil and 70.73 days in the garden soil at a CSS application rate of 25%. This was generally in agreement with the findings from the earlier studies, where *t*_1/2_ varied from 11.5 to 73 days for TCS [7,26,37]. When CSS amendment increased by 1%, *t*_1/2_ of TCS were prolonged by 2.06 days in the agricultural soil and 2.90 days in the garden soil. The *t*_1/2_ of TCS prolonged by the CSS was different from the findings in previous studies. For example, Fu et al. [10] found when biosolids amendment increased by 1%, *t*_1/2_ of TCS was prolonged by 7.5 days. However, Wu et al. [26] discovered that biosolids application had no appreciable effect on the *t*_1/2_ of TCS. The difference may be attributed to the types of biosolids used in the studies. The solid biosolids used by Fu et al. contained higher organic carbon than that used by Wu et al. and the CSS used in our experiment.

The addition of CSS increased the soil TOC content, as shown in Table 1. This would most likely promote the sorption of PPCPs by soil which could decrease its bioavailability. This thereby inhibited the degradation and prolonged the *t*_1/2_ [10]. This probably was the reason why *t*_1/2_ of TCS increased with the CSS addition rate. Moreover, the *t*_1/2_ of TCS was relatively longer in the garden soil than in the agricultural soil. A significantly positive correlation between the *t*_1/2_ of TCS and CSS application rate was observed (*R*^2^ = 0.95, *p* < 0.01). Hence, the CSS prolonged the degradation of TCS in the agricultural and garden soils. Moreover, more TCS was degraded in the agricultural soil compared to the garden soil.

**Figure 2:**
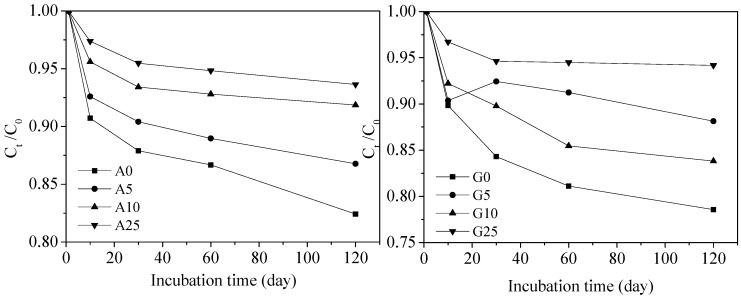
The degradation kinetics of carbamazepine (CBZ) in agricultural and garden soil (A represents agricultural soil, G represents garden soil).

By contrast, CBZ degradation was much slower compared to TCS, as shown in Figure 2. Regarding the agricultural soil, about 17.58% of CBZ was degraded in the control at the end of the incubation period, whereas in the 5% and 10% CSS treatments, about 13.23% and 8.14% of CBZ were removed, respectively. When the CSS application rate was 25%, the degradation rate was lowest, as evidenced by only 6.37% of CBZ degraded. Likewise, the amount of CBZ that was degraded decreased with the CSS addition rate with respect to the garden soil. Approximately 21.44% of CBZ was degraded in the control. When the CSS addition rate increased to 5% and 10%, the amount of CBZ removed decreased to 11.87% and 16.18%, respectively. However, only 5.82% of CBZ was removed in the 25% CSS treatment. Hence, approximately 5.82–21.43% was degraded in the agricultural and garden soil amended with a different CSS rate. The CBZ degradation decreased with CSS application rate in the two soils, and it was higher in the agricultural soil than in the garden soil, except in the control, in which the degradation rate of CBZ in agricultural soil was slightly lower than that in garden soil.

The *t*_1/2_ of CBZ had a significantly positive correlation with CSS application rate (*R*^2^ = 0.77, *p* < 0.01), see Table 2. The *t*_1/2_ of CBZ in the agricultural control and garden control was 533.19 and 407.73 days, respectively. The value reached the highest in the 25% CSS treatment, which was 1386.29 days in agricultural soil and 1732.87 days in garden soil. Our results were similar to the dissipation half-lives (355–1624 days) published by Dalkmann et al. and Kodešová et al. [23,38] and were moderately higher than the dissipation half-lives evaluated in outdoor mesocosms by Walters et al. (462–533 days) [39] and Grossberger et al. [9] (147 and >200 days). CSS amendment increased *t*_1/2_ of CBZ, increasing 1% CSS addition would prolong *t*_1/2_ by 46.60 days in the agricultural soil and 76.81 days in the garden soil. This finding was consistent with other studies. For example, Li et al. [5] found that the addition of biosolids also inhibited CBZ transformation with the estimated *t*_1/2_ at 108 days, as compared to 46 days in the same soil without the amendment.

Moreover, the *t*_1/2_ of CBZ was relatively longer in agricultural soil than in garden soil when the CSS application rates were 0% and 10%. As for the 5% and 25% CSS treatments, the opposite occurred. It is worth mentioning that the *t*_1/2_ of CBZ was longer in the 5% CSS treatment than in the 10% CSS treatment for garden soil. Although a 10% CSS treatment contained more organic carbon than a 5% CSS treatment, it seemed that the high organic carbon did not lead to inhibition of CBZ degradation in two treatments. As we mentioned above, increasing organic carbon probably enhanced sorption of PPCPs by soil, and thereby inhibited PPCPs degradation. However, soil OM likely played a dual role in the degradation of PPCPs; it served as an alternative nutrition source for the microorganisms involved in the degradation, and there was strong evidence that the total soil microbial activity increased with increasing soil OM content [40], and thereby often enhanced biotic degradation or decreased *t*_1/2_ [7,10,41,42]. This result in a 10% CSS treatment should be explained by the combined influence of these factors and is the result of the balance between different factors [42]. In general, the addition of CSS inhibited the degradation of CBZ in the agricultural and garden soils, the *t*_1/2_ of CBZ differed with soils and CSS application rate.

### 3.3. The Relationship between Soil Physicochemical Properties and Half-Life of TCS and CBZ

**Figure 3:**
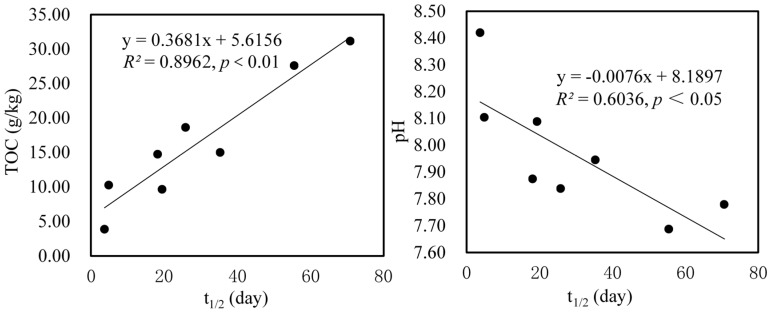
Relationship between *t*_1/2_ of TCS in two soils and soil organic carbon content and pH.

**Figure 4:**
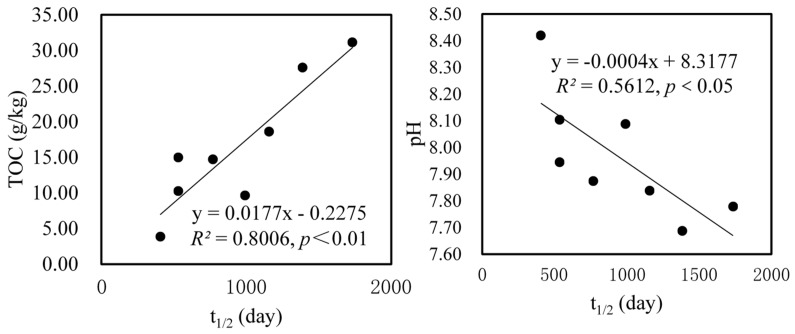
Relationship between *t*_1/2_ of CBZ and soil organic carbon content and pH in two soils.

Soil physicochemical properties, such as organic carbon content, play an important role in the degradation of PPCPs [6,7]. There was a strong positive relationship between organic carbon content and *t*_1/2_ for TCS (*R*^2^ = 0.8962, *p* < 0.01), as shown in Figure 3. Similarly, *t*_1/2_ of CBZ was significantly correlated with organic carbon content in a positive way (*R*^2^ = 0.8006, *p* < 0.01), as shown in Figure 4. The aforementioned correlation studies suggested that increasing soil organic carbon content has the potential to prolong the *t*_1/2_ of PPCPs, thereby inhibiting the degradation and removal of pollutants. This was probably ascribed to the increase of organic carbon content which promoted the sorption of PPCPs by soil and hence decreased the bioavailability of PPCPs [2,5,6,10,43]. Previous studies have confirmed that positive correlations between sorption coefficients and organic carbon content were obtained for CBZ and TCS [10,44].

In contrast with OM, soil pH was significantly negatively correlated with *t*_1/2_ of TCS and CBZ (*R*^2^ = 0.6036, *p* < 0.05 and *R*^2^ = 0.5612, *p* < 0.05), as shown in Figure 3 and Figure 4. Therefore, increasing the pH would reduce *t*_1/2_ of TCS and CBZ, and promote the degradation of TCS and CBZ. The most likely reason was that increasing the pH probably lead to the enhancement of soil microbial and enzymatic activities, and promoted PPCPs degradation. It has been confirmed that soil pH was an important factor in determining soil microbial and enzymatic activities [45,46]. Another possible reason was that PPCPs degradation was affected by its sorption behavior to soil, which was influenced by soil pH [47,48,49]. For most PPCPs, its adsorption behaviors in soil are difficult to predict, because they are controlled by interactions with specific functional groups or complicated pH-dependent speciation [7]. Various studies have documented that the adsorption of ionizable compounds was highly affected by soil pH [44,50,51,52]; like TCS, its sorption decreased with increasing pH in soil from 4 to 8 [26]. This finding indicated that an increase in the pH resulted in a decrease of the TCS sorption and an increase in the bioavailability of TCS, thus promoting the degradation of TCS, which was consistent with the result in our study. As for CBZ, its low sorption affinity to soil has been confirmed, and its sorption showed no effect within the tested pH range [43,44].

The *t*_1/2_ of TCS and CBZ varied in different soils amended with different CSS rates, and various other factors might affect their degradation behaviors. Firstly, the chemical structures and physicochemical properties (e.g., hydrophobicity and dissociation) of PPCPs might significantly affect their degradation pathways [6]. Grossberger et al. [9] observed that neutral PPCPs were more recalcitrant and persistent in soil irrigated with treated wastewater, while weakly acidic pharmaceuticals exhibited a more rapid degradation. This finding was consistent with our result that TCS was more easily degraded than CBZ. Moreover, the soil properties affected their degradation behavior. Das et al. [53] and Xu et al. [7] reported that high organic carbon inhibited the degradation of PPCPs in soil. It was also found that *t*_1/2_ of TCS and CBZ were positively correlated with organic carbon in our study, suggesting that organic carbon played an important role in PPCPs degradation.

When CSS was applied to soil, the TCS and CBZ degradation behavior was also affected by it. It was reported that biosolids amendment generally inhibited the degradation of PPCPs [5,24]; therefore, prolonging their persistence in soil. This was also confirmed in our study that the *t*_1/2_ of TCS and CBZ was positively related to the CSS addition amount (*R*^2^ = 0.95, *p* < 0.01 and *R*^2^ = 0.77, *p* < 0.01). The inhibitory effect of CSS was probably ascribed to organic carbon, which was found in various studies [5,6,7,10]. The addition of CSS increased the organic carbon in soil, which might lead to the enhancement of sorption of PPCPs to soil and the decrease of the bioavailability of PPCPs, thus inhibiting PPCPs degradation. Another possible reason was heavy metals introduced by CSS, which might affect PPCPs degradation. This was not involved in this study, but previous studies have reported that heavy metals like Cu, Zn, and Pb might have an adverse effect on microbial activities [54,55], and thereby inhibit the PPCPs degradation. Nevertheless, it could not be ignored that biosolids might also contribute to the degradation of PPCPs by bringing in a large number of microorganisms and rich material available for microbial use [5,6]. Hence, the effect of CSS on PPCPs degradation was the balance of various factors. Moreover, the inhibitory effect of CSS played a dominant role in the degradation of TCS and CBZ in most treatments in our study.

The use of compost sewage sludge should be strictly controlled. Once it is applied to soil, PPCPs introduced by it may eventually lead to the deterioration of the soil and groundwater quality. Hence, the use amount and purpose of land use should be carefully considered. Moreover, when assessing ecological risks or human exposure of TCS and CBZ, the increased persistence due to biosolids amendment should be considered.

### 3.4. Correlations between Soil Enzymes and C_t_/C_0_ of TCS and CBZ

**Figure 5:**
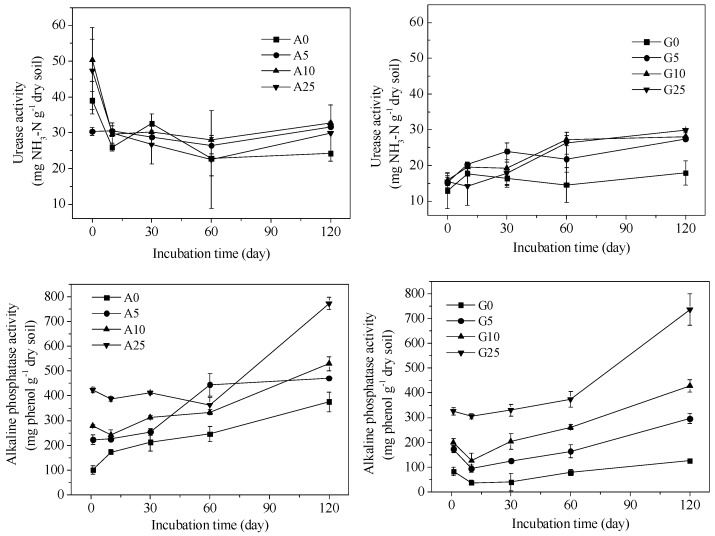
The soil urease and alkaline phosphatase activities in agricultural and garden soil amended with different CSS application rates.

**Figure 6:**
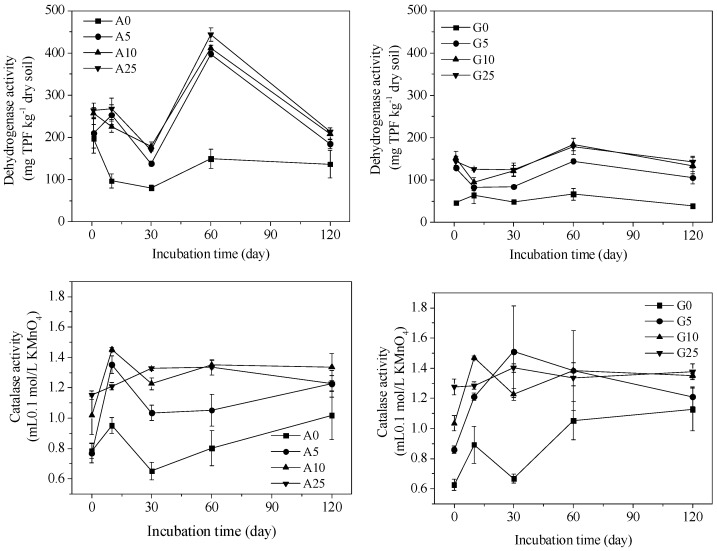
The soil dehydrogenase and catalase activities in agricultural and garden soil amended with different CSS application rates.

Soil enzyme activities were greatly affected by CSS, as shown in Figure 5 and Figure 6. The urease activity in agricultural soil reached the highest value at the initial incubation time, decreased on Day 10, and remained unchanged during the following incubation period. In the case of the garden soil, the urease activity exhibited a growing trend with incubation time. In general, the urease activity in the agricultural soil was higher than in the garden soil. The application of 5% and 10% CSS resulted in a higher urease activity compared to the 0% and 25% CSS application treatments. Alkaline phosphatase activity in the agricultural and garden soils generally increased with incubation time and CSS application rate. Moreover, the alkaline phosphatase activity in the agricultural soil was relatively higher than in the garden soil. As for dehydrogenase activity, it also increased with CSS application rate. Moreover, the dehydrogenase activity was also higher in the agricultural soil than in the garden soil. However, it reached the highest values on the 60th day. The catalase activity value fluctuated before Day 60 but remained unchanged in the latter time. There was no significant difference in the catalase activity between the two soils. The addition of CSS increased the catalase activity compared to the control soil.

Correlation studies showed negative relationships between the C_t_/C_0_ of TCS and CBZ and soil catalase, dehydrogenase, and alkaline phosphatase activities in almost all different CSS application treatments, see Table 3 There were significantly negative relationships between alkaline phosphatase activity and C_t_/C_0_ of TCS in G25 treatment (*r* = −0.936, *p* < 0.05) and CBZ in A0 treatment (*r* = −0.926, *p* < 0.05). For urease, in the agricultural soil, it showed a positive relationship with the C_t_/C_0_ of TCS and CBZ, while in the garden soil a negative correlation between urease activity and C_t_/C_0_ of TCS and CBZ was observed. A significantly negative correlation between the urease activity and the C_t_/C_0_ was found in G10 and G25 for TCS (*r* = −0.983, *p* < 0.01 and *r* = −0.914, *p* < 0.05, respectively) and in G10 for CBZ (*r* = −0.921, *p* < 0.05).

## 4. Conclusions

TCS was degraded totally during the incubation period, while only approximately 5.82–21.43% of CBZ was degraded. The degradation of TCS and CBZ decreased with CSS application rate in almost all treatments except for the garden soil amended with 10% CSS. This finding suggested that the CSS addition inhibited the degradation of TCS and CBZ and prolonged their *t*_1/2_. Correlation studies showed a significantly positive relationship between *t*_1/2_ of TCS and CBZ and TOC, while a significantly negative relationship between *t*_1/2_ and pH was observed. As for the role of enzyme activities in the degradation of TCS and CBZ, the alkaline phosphatase showed a significantly negative relationship with the C_t_/C_0_ of TCS in G25 and CBZ in A0 treatments. The urease activity was significantly negatively correlated with the C_t_/C_0_ of TCS in G10 and G25 treatments and CBZ in G10 treatment.

## Figures and Tables

**Table 1:** Physicochemical properties of the agricultural soil, garden soil, and composted sewage sludge (CSS).

Soil Types	pH	TOC ^a^ (g/kg)	CEC ^b^ (cmol(+)/kg)	EC ^c^ (μs/cm)	Clay (%)	Slit (%)	Sand (%)
A0	8.16	10.63	6.37	345	4.7	17.3	78.0
A5	7.87	14.70	7.45	674	5.6	20.2	74.2
A10	7.83	19.27	9.69	928	5.7	22.2	72.1
A25	7.73	26.97	11.12	1544	5.4	24.7	69.8
G0	8.41	3.72	17.41	157	12.2	34.7	53.1
G5	8.05	9.73	23.24	490	11.5	33.6	54.9
G10	7.95	15.10	18.42	744	11.7	34.6	53.7
G25	7.75	32.23	19.27	1424	10.2	34.8	55.0
CSS	7.50	148.67	45.4	5 373	6.0	32.5	61.5

^a^ TOC: total organic carbon; ^b^ CEC: cation exchange capacity; ^c^ EC: electrical conductivity.

**Table 2:** Data of the exponential decay model for TCS and CBZ.

Soil + Treatments	CSS Addition Rate (%)	TCS		CBZ	
		k ^a^ (1/d)	*t*_1/2_ (d)	k (1/d)	*t*_1/2_ (d)
A0	Control	0.1429	4.85	0.0013	533.19
A5	5	0.0381	18.19	0.0009	770.16
A10	10	0.0268	25.86	0.0006	1155.24
A25	25	0.0125	55.45	0.0005	1386.29
G0	Control	0.1873	3.70	0.0017	407.73
G5	5	0.0357	19.42	0.0007	990.21
G10	10	0.0196	35.36	0.0013	533.19
G25	25	0.0098	70.73	0.0004	1732.87

^a^ k is the first order degradation rate constant (1/day for the biodegradation tests).

**Table 3:** The correlations between enzyme activities and C_t_/C_0_ of TCS and CBZ.

**C_t_/C_0_ of TCS**	**Urease**	**Catalase**	**Dehydrogenase**	**Alkaline Phosphatase**
A0	0.792	−0.105	0.695	−0.762
A5	0.352	−0.335	−0.264	−0.894
A10	0.721	−0.588	−0.340	−0.701
A25	0.580	−0.396	0.077	−0.778
G0	−0.709	−0.626	−0.213	0.050
G5	−0.842	−0.676	−0.185	−0.513
G10	−0.983 **	−0.568	−0.410	−0.744
G25	−0.914 *	−0.654	−0.211	−0.936 *
**C_t_/C_0_ of CBZ**	**Urease**	**Catalase**	**Dehydrogenase**	**Alkaline phosphatase**
A0	0.828	−0.339	0.532	−0.926 *
A5	0.160	−0.655	−0.130	−0.755
A10	0.856	−0.680	−0.072	−0.620
A25	0.830	−0.679	−0.044	−0.508
G0	−0.601	−0.761	−0.006	−0.296
G5	−0.870	−0.646	0.356	−0.258
G10	−0.921 *	−0.688	−0.160	−0.657
G25	−0.684	−0.809	−0.172	−0.473

* Significance level of *p* < 0.05. ** Significance level of *p* < 0.01.
